# Supplementary material for: IL-15 sustains IL-7R-independent ILC2 and ILC3 development
Source: Nat Commun. 2017 Mar 31;8:14601. doi: 10.1038/ncomms14601 (PMC5380969; doi:10.1038/ncomms14601)
Supplement: Supplementary Information — Supplementary Figures and Supplementary Tables [file ncomms14601-s1.pdf]

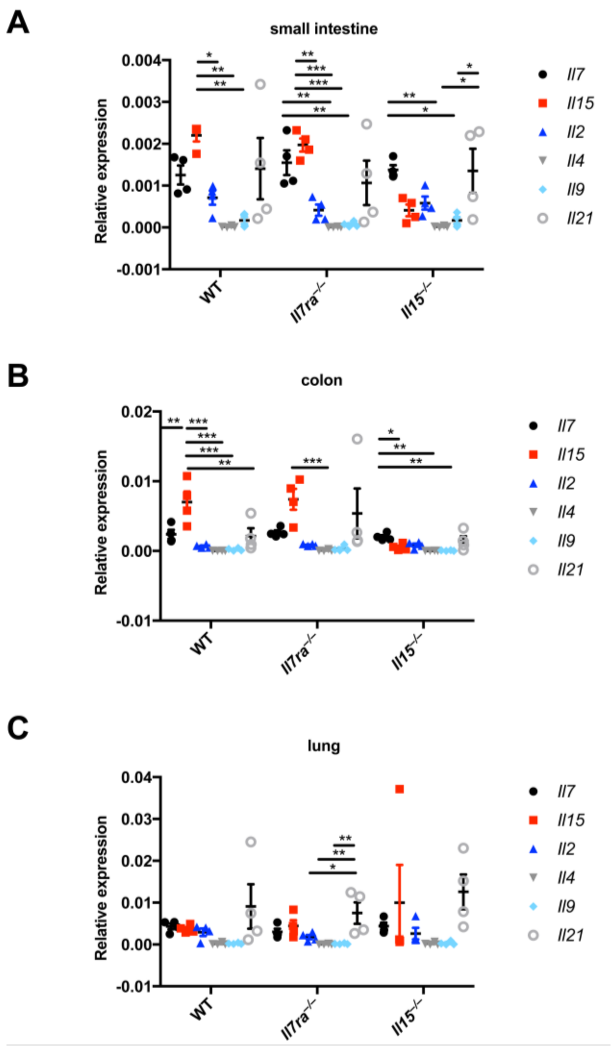

Supplementary Figure 1. mRNA expression of γ_c cytokines.

(**A-C**) γ_c cytokine mRNA were measured in the (**A**) small intestine, (**B**) colon, and (**C**) lung of WT, *Il7ra*$^{-/-}$, and *Il15*$^{-/-}$ mice. Cytokine expression was calculated relative to β-actin. Expression levels of different γ_c cytokines were compared between organs for each genotype. Error bars mean ± SEM. *p<.05, **p<.01, ***p<.001, one-way ANOVA with Tukey's test for multiple comparisons.

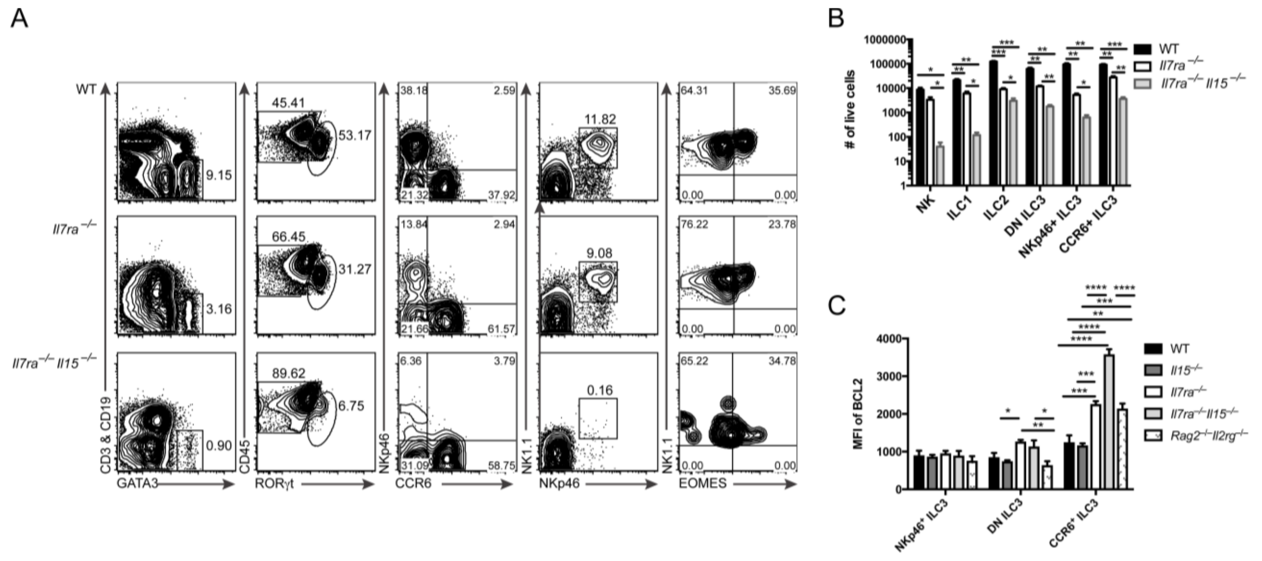

Supplementary Figure 2. Extended analyses of *Il7ra*$^{-/-}$*Il15*$^{-/-}$ mice.

(A) Representative flow plots of siLP ILCs, as gated in **1A**. (**B**) Total number of indicated ILC subsets from the siLP. (**C**) MFI of Bcl-2 measured by flow cytometry between ILC3 subsets. Error bars mean ± SEM. Data represent (**A-B**) n = 5 mice or (**C**) n=4 mice per genotype from 2 independent experiments. *p<.05, **p<.01, ***p<.001, ****p<.0001, one-way ANOVA with Tukey's test for multiple comparisons.

Supplementary Table 1. Gene Expression Differences Between ILC3 subsets.

**NKp46$^+$ ILC3 genes**	FC: NKp46$^+$/DN	FC: CCR6/DN
Slc6a20a	9.20	0.53
Nmbr	8.99	0.74
Klri2	7.37	1.45
Slamf7	6.30	0.70
Xcl1	6.12	0.50
Klrb1c	5.01	1.04
Klrb1f	4.69	1.24
Sulf2	4.03	0.90
Cd244	3.84	0.80
Tbx21	3.47	0.49
Gda	3.42	0.20
Pydc3	3.06	0.39
Klrb1a	3.00	1.40
Cdk14	2.81	0.35
Abi3bp	2.33	0.96
Dusp2	2.32	0.73
Ccr9	2.32	0.15
Tmem37	2.24	0.70
Myc	2.10	1.00

**DN ILC3 genes**	FC: DN/ NKp46$^+$	FC: DN/ CCR6
Il17rb	4.48	2.49
Chchd5	3.08	2.24
Hes1	2.95	2.49
Znrf3	2.52	2.10
Arl5c	2.23	2.45
Zcchc18	2.02	2.10

**NKp46$^+$& DN ILC3 genes**	FC: DN/ NKp46$^+$	FC: DN/ CCR6
Ifng	0.59	15.90
Ctsw	0.58	9.82
Nkg7	0.41	6.89
Ccr9	0.43	6.87
Cxcr3	0.72	6.38
Il2	0.38	4.95
Gda	0.29	4.93
Eps8l3	0.72	4.10
Csf2	1.26	4.01
Serpinb9	0.83	3.62
Dppa2	1.11	3.46
Ryk	1.14	3.21
Abcg3	0.88	3.06
Cdk14	0.36	2.89
Pcp4	1.06	2.68
Nt5e	0.84	2.63
Socs2	0.83	2.53
Ikzf3	0.71	2.52
1810011H11Rik	0.68	2.50
Skap1	0.87	2.46
Itih5	0.92	2.46
Ccnd2	0.90	2.44
Cd226	0.87	2.43
Glcci1	0.60	2.41
4930503L19Rik	0.56	2.35
Lef1	0.80	2.21
Gimap3	0.95	2.15
Slc27a6	0.65	2.11
Lgals1	0.57	2.11
Dusp6	0.96	2.07
Adrb2	0.94	2.06
Tbx21	0.29	2.06
Xcl1	0.16	2.02
Fam63b	0.92	2.02

**DN & CCR6$^+$ ILC3 genes**	FC: DN/ NKp46$^+$	FC: CCR6/DN
Cd3e	16.81	0.38
Cd3d	16.50	0.34
Il17a	10.89	0.66
Cd40lg	7.70	0.69
Gria3	4.56	0.90
Ret	4.30	1.32
Ly86	4.09	3.72
Bmp2	3.98	4.34
Nrp1	3.97	3.11
Npnt	3.92	1.80
Npl	3.64	0.90
Hmgn3	3.40	2.53
Cntn1	3.28	1.98
Itk	3.18	1.58
Dll1	3.02	1.35
Khdrbs3	3.02	1.82
Ramp3	2.51	0.86
4930506M07Rik	2.33	1.01
Pdcd1	2.11	1.95
Sdc4	2.03	1.19

CCR6+ ILC3 genes	FC: DN/ NKp46+	FC: CCR6/DN
S100g	0.71	13.37
Dmbt1	1.10	11.62
Ccdc88a	1.27	10.22
Plb1	1.02	9.16
H2-Ab1	0.98	9.11
Cd4	1.26	8.95
Parvb	1.26	8.39
Gm10802	0.98	8.11
Alcam	0.90	7.49
Dscam	1.07	7.09
Maged1	1.03	7.08
Fut4	0.92	5.53
Olfm4	1.05	5.42
Mef2c	1.18	5.14
BC018473	0.92	5.03
Zc3h12c	1.70	5.01
Fermt2	1.68	4.97
H2-Eb1	1.03	4.97
Wfdc18	1.27	4.85
Serpine2	1.30	4.64
Bmp2	3.98	4.34
Il6st	1.37	4.04
Mmp23	0.58	3.95
Cacna1g	1.73	3.93
Mapk10	1.23	3.90
Ly86	4.09	3.72
Cd81	6.45	3.18
Nrp1	3.97	3.11
Marcks	1.06	3.10
Dennd3	1.27	2.98
Tmem206	0.92	2.80
Rfx3	0.98	2.80
Il17f	1.80	2.79
Fam189a1	1.04	2.76
Ctsl	1.34	2.74
Cx3cl1	2.71	2.72
Camsap2	0.82	2.67
Dnajc13	0.81	2.64
Pacsin1	1.47	2.59
Hmgn3	3.40	2.53
Fam109b	1.04	2.48
Mfsd7b	1.09	2.33
Tm9sf2	1.52	2.27
Bst2	1.44	2.25
Pkp4	1.57	2.20
Tox2	1.62	2.20
Maml3	1.22	2.16
Sypl	1.04	2.14
Myof	1.33	2.13
Ern1	1.21	2.10
D430042O09Rik	1.11	2.09
Nxpe3	1.30	2.05
Col4a3bp	1.08	2.04

Supplementary Table 2. Flow Cytometry Reagents.

Antibody	Clone	Dilution	Manufacturer
anti-$\alpha_4\beta_7$	DATK32	1:200	eBioscience
Anti-B220	RA3-6B2	1:200	BioLegend
anti-Bcl-2	10C4	1:100	eBioscience
anti-CCR6	140706	1:200	BD Bioscience
anti-CD11b	M1/70	1:200	BD Bioscience
anti-CD11c	N418	1:200	eBioscience
anti-CD122	TM-β1	1:100	BioLegend
anti-CD127	A7R34	1:100	BioLegend
anti-CD19	ebio1D3	1:200	eBioscience
anti-CD19	1D3	1:200	BD Bioscience
anti-CD244	eBio244F4	1:200	eBioscience
anti-CD25	PC61	1:100	BioLegend
anti-CD27	LG.7F9	1:200	eBioscience
anti-CD3ε	145-2C11	1:200	eBioscience/BD Bioscience
anti-CD4	GK14	1:200	BioLegend
anti-CD45	30-F11	1:200	eBioscience
anti-CD5	53-7.3	1:200	eBioscience
anti-CD8a	53-6.7	1:200	eBioscience
anti-CD90.2	30-H12	1:200	eBioscience
anti-EOMES	Dan11mag	1:100	eBioscience
anti-Flt3	A2F10	1:100	eBioscience
anti-GATA3	L50-823	1:100	BD Bioscience
anti-IL-13	eBio13A	1:100	eBioscience
anti-IL-22	1H8PW8R	1:100	eBioscience
anti-IL-5	TRFK5	1:100	BD Bioscience
anti-IL17a	TC11-18H10	1:100	BD Bioscience
anti-IL25R	MUNC33	1:200	eBioscience
anti-Ki67	SolA15	1:300	eBioscience
anti-KLRG1	2F1	1:200	eBioscience
anti-Ly6G	1A8	1:200	BioLegend
anti-NK1.1	PK136	1:200	eBioscience
anti-NKp46	29A1.4	1:100	eBioscience
anti-NKp46	CS96	1:100	Colonna Lab
anti-RORγt	AFKJS-9	1:100	eBioscience
anti-ST2	RMST2-2	1:100	eBioscience
SAV BV421		1:200	BD Bioscience
SAV Pe-Cy7		1:200	eBioscience

Supplementary Table 3. RT-PCR Primer Sequences.

Primer	Direction	Sequence
Actb	F	5’-GACGGCCAGGTCATCACTATTG-3’
Actb	R	5’-AGGAAGGCTGGAAAAGAGC-3’
Il2	F	5’-TGAGCAGGATGGAGAATTACAGG-3’
Il2	R	5’-GTCCAAGTTCATCTTCTAGGCAC-3’
Il4	F	5’-ATCATCGGCATTTTGAACGAGG-3’
Il4	R	5’-TGCAGCTCCATGAGAACACTA-3’
Il7	F	5’-TTCCTCCACTGATCCTTGTTCT-3’
Il7	R	5’-AGCAGCTTCCTTTGTATCATCAC-3’
Il9	F	5’-CATCAGTGTCTCTCCGTCCCAACTGATG-3’
Il9	R	5’-GATTTCTGTGTGGCATTGGTCAG-3’
Il15	F	5'-GTTCTGGATGGATGGCAGCT-3'
Il15	R	5'-ACATGAATGCCAGCCTCAGT-3'
Il21	F	5’-GGACCCTTGTCTGTCTGGTAG-3’
Il21	R	5’-TGTGGAGCTGATAGAAGTTCAGG-3’
